# Development and Validation of a Novel LC-MS/MS Method for a TDM-Guided Personalization of HSCT Conditioning with High-Dose Busulfan in Children

**DOI:** 10.3390/biomedicines11020530

**Published:** 2023-02-11

**Authors:** Alessia Cafaro, Federica Pigliasco, Giammarco Baiardi, Sebastiano Barco, Manuela Stella, Roberto Bandettini, Francesca Mattioli, Maura Faraci, Giuliana Cangemi

**Affiliations:** 1 Chromatography and Mass Spectrometry Section, Central Laboratory of Analysis, IRCCS Istituto Giannina Gaslini, 16147 Genoa, Italy; 2 Pharmacology & Toxicology Unit, Department of Internal Medicine, University of Genoa, Viale Benedetto XV 2, 16132 Genoa, Italy; 3 Clinical Pharmacology Unit, EO Ospedali Galliera, Mura delle Cappuccine, 14, 16128 Genoa, Italy; 4 Hematopoietic Stem Cell Unit, Department of Pediatric Hematology and Oncology, IRCCS Istituto Giannina Gaslini, 16147 Genoa, Italy

**Keywords:** busulfan, hematopoietic stem cell transplantation, drug monitoring, LC-MS/MS, pediatrics

## Abstract

Personalization of busulfan (Bu) exposure via therapeutic drug monitoring (TDM) is recommended for patients treated with high-dose conditioning regimens. Several laboratories’ developed methods are available in the literature with a lack of standardization. The aim of this study is to develop a new standardized LC-MS/MS method and validate it according to the international ICH M10 (EMA) guidelines. Our method is based on rapid protein precipitation from 50 µL plasma followed by separation on a reversed-phase C-18 UHPLC column after the addition of deuterated internal standard and has been tested on real samples from pediatric patients treated with myeloablative conditioning regimens, including Bu, before autologous or allogeneic hematopoietic stem cell transplantation (HSCT). The validated LC-MS/MS method is linear over wide concentration ranges (125–2000 ng/mL), accurate, and reproducible in the absence of matrix effects, allowing for the specific and rapid quantification of Bu and allowing next-dose recommendations to be made in a timely fashion to answer clinicians’ needs. Given the lack of data on the stability of Bu in real clinical samples, stability was assessed both on quality controls and on real samples to set up a robust protocol in real-life conditions. This novel LC-MS/MS method is suitable for application to the TDM-guided personalization of conditioning treatments with high-dose busulfan in pediatric patients undergoing HSCT.

## 1. Introduction

Busulfan (Bu) is a non-specific cell cycle alkylating antineoplastic agent from the class of alkyl sulfonates, widely used for conditioning regimens before hematopoietic stem cell transplantation (HSCT) in combination with other cytotoxic drugs, such as cyclophosphamide or melphalan. Its mechanism of action is based on the alkylation of DNA by carbonium ions, which are rapidly formed following systemic absorption [1,2,3,4], thus producing guanine-adenine intra-strand cross-linkages, which leads the cells to apoptosis [5]. Since the 1970s, high-dose Bu therapy has been used to replace total body irradiation as a myeloablative preparatory regimen before HSCT [6]. Oral Bu administration suffers from erratic gastrointestinal absorption and hepatic first-pass effects, affecting inter- and intra-patient variability in pharmacokinetics parameters (PKs), particularly in children [7]. Intravenous (IV) Bu tends to then be chosen preferentially on the basis of concerns about PK variability and the narrow therapeutic index of Bu [8]. Dose adjustment of Bu based on therapeutic drug monitoring (TDM) to bring exposures within established therapeutic ranges has been shown to reduce the risk of adverse events and improve the therapeutic efficacy of the conditioning treatment [5], and it is, therefore, the current standard practice adopted in many pediatric centers. The initial IV Bu dose should be based on the European Medicines Agency (EMA) nomogram for children with a target area under the plasma concentration-time curve (AUC) per administration of 1125 μmol/L × minute (range 900 to 1350 ± 5% μmol/L × minute), which is equivalent to a target concentration at steady state (C_SS_) of 770 ng/mL (range 650 ng/mL to 1026 ng/mL) [8]. The personalization of Bu dose to a target exposure is based on TDM: from the measured Bu plasmatic concentrations, patient’s PKs are estimated by pharmacokinetic principles and then used for the appropriate adjustment of subsequent Bu doses [7,8,9,10,11,12]. Bu TDM-guided dosing is mandatory in children receiving high-dose Bu before allogeneic HCT to lower the risk of dose-limiting toxicity and improve patients’ clinical outcomes [8]. The regulatory agency-suggested Bu therapeutic AUC window is between 900 and 1500 µmol/L × minute per administration (equivalent to 3.90–6.16 mg/L × hour) [13]. The standard clinical practice of HCST conditioning treatment with a high Bu dose requires the collection of at least 3 blood samples for AUC proper estimation and adequate subsequent Bu-dose individualization. 

The availability of robust analytical methods able to quantify Bu with rapid turn-around times is, therefore, crucial in pediatric clinical laboratories. Even if the TDM of Bu is crucial for decision making on the next dose to be administered, to our knowledge, no certified liquid chromatography coupled to tandem mass spectrometry (LC-MS/MS) methods for Bu quantification in plasma are available on the market for in vitro diagnostic use. Several laboratory-developed tests are available in the literature, presenting heterogeneous extraction and analytical protocols and confirming a lack of standardization. Moreover, the lack of data on the stability of Bu in patients’ samples in real-life conditions makes it difficult to set up robust protocols for TDM.

Chromatographic methods, such as gas chromatography coupled to mass spectrometry (GC-MS) or high-performance liquid chromatography (HPLC), have been adopted since the 1980s [14,15,16,17]. However, nowadays, LC-MS/MS has become the gold standard for the quantitation of small molecules, being able to guarantee higher specificity, accuracy, and productivity compared with HPLC and GC-MS methods [18,19]. Thanks to the improved instrumentation, the availability of several clinical applications and the widespread use of commercial kits, the use of LC-MS/MS in clinical laboratories has become considerably routinized over the past 20 years [18,19]. In this manuscript, we show the development of a standardized LC-MS/MS method and its validation according to the updated international guidelines (ICH guideline M10) [20] to support the treatment of children undergoing myeloablative chemotherapy prior to HSCT with the TDM-guided dose adjustment of high-dose Bu.

## 2. Materials and Methods

### 2.1. Chemicals and Reagents

High-performance liquid chromatography (HPLC)-grade methanol and acetonitrile (ACN) were purchased from Sigma-Aldrich Srl (Milan, Italy). MS-grade water (MilliQ, manufacture, Milan, Italy) was produced with a Milli-DI system coupled with a Synergy 185 system by Millipore (Milan, Italy). Ammonium acetate (ref. 431311), zinc sulfate heptahydrate and LC–MS/MS-grade formic acid (ref. 607001000) were purchased from Sigma (Milan, Italy). All reagents had 98% purity. All solutions were prepared with HPLC-grade water obtained from a Milli-Q Plus water purification system. HPLC mobile phases were filtered using Millipore membrane filters (0.45 µm) (Millipore, Vimodrone, Italy).

### 2.2. Preparation of Calibration Standards and Stock Solutions 

Stock solutions of Bu 1000 µg/mL (B-094) and Bu-d8 100 µg/mL (B-095) in ACN were provided by Merck Life Science S.r.l., Milano, Italy). Working solutions of Bu (500 µg/mL) and Bu-d8 (3 µg/mL) were obtained by diluting the stock solution with an appropriate ACN volume. The Bu working solution (500 µg/mL) was diluted with an appropriate volume of blank plasma to prepare calibrators and QC samples, which were then divided into 50 µL aliquots. The six calibrators concentrations were 2000 ng/mL, 1000 ng/mL, 800 ng/mL, 500 ng/mL, 250 ng/mL, and 125 ng/mL. The four quality control (QC) concentrations (LLOQ, QC low, QC medium, and QC high) were 125 ng/mL (LLOQ), 300 ng/mL (QC low), 600 ng/mL (QC medium), and 900 ng/mL (QC high). 

### 2.3. Sample Preparation 

Sample preparation was performed as follows: 50 µL of plasma (calibrators, QCs and patient samples) + 10 µL of IS working solution (3 µg/mL) + 440 µL of 0.1% *v*/*v* formic acid in ACN. After vortexing, samples were centrifuged at 14,000× *g* for 10 min at 4 °C, and the obtained eluate was then transferred into autosampler vials and injected into the LC-MS/MS system for analysis.

### 2.4. Chromatographic Conditions 

The chromatographic run was performed on Ultimate 3000 UHPLC Dual Gradient Pumps (Thermo Fisher Scientific, Milan, Italy) using an Acquity UPLC BEH C18 LC column (2.1 mm × 100 mm, i.d. 1.7 µm, Waters SpA, Milan, Italy). The flow rate was set at 400 µL/min. The mobile phases were ammonium acetate 5 mM and formic acid 0.1% *v*/*v* in water (phase A) and formic acid 0.1% *v*/*v* in ACN (phase B). The concentration of the phases in gradient (%*v*/*v*) is shown in Table 1.

### 2.5. MS/MS Conditions 

Tandem mass spectrometry detection was performed using a TSQ Quantiva triple quadrupole system (Thermo Fisher Scientific, Milan, Italy) equipped with an electrospray ionization source (ESI). The ESI operated in positive ion mode for both Bu and IS (spray voltage at 4500 V). Nitrogen was used as a nebulizer and auxiliary gas, set at 40 and 5 arbitrary units, respectively. The vaporizer and capillary temperatures were both set at 350 °C. Argon (pressure of 2.5 mTorr) was used as the collision gas. The specific ions’ transitions of Bu and its deuterated IS were detected by single-reaction monitoring (SRM): 264.029→151.071 for Bu and 272.068→159.125 for Bu-d8. 

### 2.6. Method Validation 

For method validation purposes, blank samples were obtained from healthy adult volunteers who were not under Bu treatment. The method validation was performed according to the recently approved ICH guideline M10 [20].

#### 2.6.1. Selectivity and Specificity

Samples from six healthy volunteers not taking drugs and from 12 patients on Bu therapy were analyzed to assess selectivity and specificity. For each batch, one sample spiked with Bu at LLOQ and one sample spiked with IS were analyzed by the same method. For the investigation of selectivity and specificity in haemolysed matrices, one haemolysed matrice was obtained by spiking a blank plasma sample with haemolysed whole blood (2% *v*/*v*) to generate a haemolysed sample. Responses attributable to interfering components were considered acceptable if there were no more than 20% of the analyte response to the LLOQ and no more than 5% of the IS response in the LLOQ sample for each matrix.

#### 2.6.2. Matrix Effect and Extraction Recoveries

The matrix effect was evaluated by analyzing 3 replicates of QC low and high; each was prepared using a matrix from 6 different healthy volunteers not consuming drugs. For each individual matrix evaluated, the matrix effect was determined by comparing the peak area of QC low and QC high after extraction to the peak area of pure solutions at the same concentration, according to B.K. Matuszewski et al. [21]. Recovery was determined by comparing the peak area of the analytes spiked before extraction to the peak area of the analytes spiked after extraction.

#### 2.6.3. Calibration Curve and Range

The calibration curve consists of six concentration levels, and it was validated in the range of 125–2000 ng/mL. Linearity was assessed by analyzing the calibration curve three independent times. A weighting factor of 1/x was used to adjust the peak area ratio of analyte/IS vs. the analyte concentration of the calibrator. Accuracy was considered acceptable if it was within ±15% of the theoretical value for each calibrator, except for LLOQ (±20%).

#### 2.6.4. Precision and Accuracy 

The four QCs (LLOW, low QC, medium QC, and high QC) were analyzed five times on three different days to assess within-run and between-run accuracy and precision within and between runs. Accuracy was evaluated as the mean relative error (expressed as a percentage), and precision was evaluated as the coefficient of variation (CV%). Precision and accuracy results were considered acceptable if ±15% for each level and ±20% for LLOQ.

#### 2.6.5. Carry-Over 

The presence of carry-over was investigated by analyzing blank samples in triplicate after the injection of the highest calibration standard. According to ICH guideline M10, to consider carry-over negligible, the signal in the blank sample following the upper standard was required to be less than 20 percent of the LLOQ and 5 percent for the IS.

#### 2.6.6. Dilution Integrity

To evaluate dilution integrity, two spiked samples were prepared, with respective Bu concentrations of 2500 and 3500 ng/mL (both higher concentrations of ULOQ). Each diluted sample was analyzed five times. The mean accuracy of the dilution QCs was considered acceptable within ±15% of the nominal concentration, and the precision (%CV) was considered acceptable if it did not exceed 15%.

#### 2.6.7. Stability 

As suggested by ICH guideline M10, samples were considered stable if the percentage difference, calculated as the ratio between the concentration measured at each sampling point and the initial concentration, was lower than 15%.

- *Freeze-thaw stability in matrix*

To assess the impact of repeatedly removing samples from frozen storage, the stability of Bu was analyzed after three cycles of freezing and thawing. Low and high QCs were thawed and analyzed according to the same procedures as the study samples. QCs were kept frozen for at least 12 h between the thawing cycles. QCs for freeze-thaw stability were assessed using freshly prepared calibration standards and QCs.

- *Stability in spiked samples*

The long-term stability of Bu was assessed by analyzing three replicates of QC low and QC high stored at −20 °C and −80 °C for 4 weeks. 

The short-term stability of Bu in spiked samples was investigated within 24 h by analyzing three replicates of QC low and QC high stored at room temperature (25 ± 2 °C), at 4 ± 3 °C, and at −20 °C.

- *Autosampler stability*

Autosampler stability was assessed by analyzing samples after 24 h.

- *Stability of the analyte in whole blood*

The stability of Bu in whole blood was evaluated on real samples by the triplicate analysis of two different patients’ samples stored at room temperature (25 ± 2 °C) and at 4 ± 3 °C for 24 h.

- *Stability of the analyte in real samples*

The stability of Bu in real samples was evaluated by the analysis in triplicate of two patients’ samples stored at room temperature (25 ± 2 °C), at 4 ± 3 °C, and at −20 °C for 24 h.

### 2.7. Human Samples 

The study was conducted in accordance with the ethical standards of the Institutional and National Research Committee and with the 1975 Helsinki Declaration revised in 2013. Written informed consent was obtained from all patients or legal representatives at the time of admission to use clinical data for research purposes, following the privacy policy of IRCCS Istituto Giannina Gaslini, Genoa, Italy. 

The suitability of the developed method was tested on plasma samples obtained according to the standard clinal practice of Bu conditioning treatment from 12 pediatric patients who were candidates for HCST at Giannina Gaslini Tertiary Care Pediatric Hospital (Genoa, Italy) (age 1–17 years, 11 M, 1 F). Blood samples were collected from each patient before the administration of the conditioning treatment with Bu 0, 2, 3, 4 and 6 h after the start of the infusion. Plasma was obtained from peripheral blood collected in tubes with EDTA K3 anticoagulant by centrifuging at 4000× *g* for 5 min. Plasma samples were frozen at −20 °C until analysis.

### 2.8. Pharmacokinetic Analysis

PK parameters (PKs) were determined by a noncompartmental method using an appropriate model of 8.3.5 Phoenix WinNonlin Professional Edition (Certara France Sarl). The main PKs calculated for each patient were the area under the plasma concentration–time curve from the time of dosing to the time of the last measurable post-dose concentration (AUC_last_), the area under the curve extrapolated to infinity (AUC_inf_), and the average plasma drug concentration at steady-state (C_ss_). AUC_last_ was calculated by the linear trapezoidal method with linear interpolation between measured data points and AUC_inf_ by adding the ratio of the last measurable plasma concentration value (C_last_) and constant elimination rate (K_el_); C_ss_ was calculated by the ratio of AUC_inf_ of a single dose and dosing interval (tau). K_el_ was estimated from the slope of the terminal phase of the plasma concentration versus the time curve by log-linear regression analysis. Estimations of K_el_ and parameters derived from them (i.e., terminal half-life, t_1/2_) were considered to be reliable only when the regression included at least three midpoints’ data in the elimination phase of the plasma concentration–time curve with r^2^ > 0.80.

### 2.9. External Quality Assessment

Our laboratory participated in an external quality assessment (EQA) program for the measurement of Bu in plasma organized by SKML (Radboud University, Mercator 2, Toernooiveld 300, Nijmegen, The Netherlands). Two samples were tested in May 2022, and two samples were tested in November 2022.

## 3. Results

### 3.1. Method Development 

Six different sample preparation protocols were tested on 5 replicates of QC low and QC high (Table 2). The adopted extraction procedure described in Section 2.4 (Materials and Methods) was protocol number six. During the method development process, protein precipitation was initially performed by adding 150 µL acetonitrile to a 50 µL plasma sample. During the validation process, both QCs and real samples were analyzed in triplicate in 3 different analytical sessions. Although the results obtained for QCs were acceptable in terms of intra-day and inter-day reproducibilities (CV < 15%), those obtained on real samples ranged from 10% to 40% (mean = 17%). Two additional sample preparation protocols, including a higher volume of the organic phase, were then tested, obtaining an improvement of reproducibilities (10% and 4% for protocols 5 and 6, respectively), therefore meeting the criteria of ICH M10 guidelines. Therefore, it was decided to adopt protocol 6, which gave extraction recovery results that were acceptable and equal to those of protocol 4 but allowed acceptable reproducibility in real samples.

Three LC columns were tested: (1) a Thermo Scientific Accucore Polar Premium column (50 mm × 2.1 mm, i.d. 2.6 µm, Thermo Fisher Scientific, Milan, Italy), (2) a Hypersil GOLD aQ (50 × 2.1 mm, i.d. 1.9 µm, Thermo Fisher Scientific, Milan, Italy) and (3) an Acquity UPLC BEH C18 (2.1 mm × 100 mm, i.d. 1.7 µm, Waters SpA, Milan, Italy). The coloumn which gave the best results in terms of resolution, symmetry and peak classification as determined by the default parameters of the peak suitability software (Supplementary Materials Figure S1, Tables S1 and S2) is column number three. Column 3’s retention time obtained for Bu and Bu-d8 was 2.71 min (±0.10).

### 3.2. Method Validation

The results are derived from the measured concentrations of the validation samples and were acceptable according to the ICH Guideline M10 [20].

#### 3.2.1. Selectivity and Specificity

**Figure 1:**
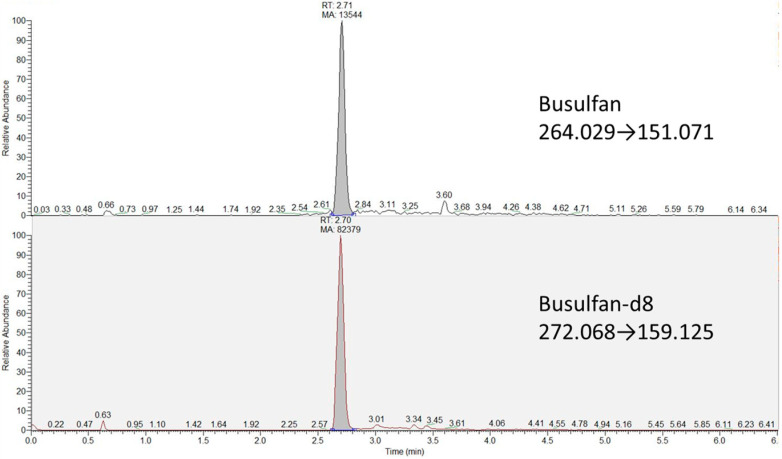
Chromatograms obtained from the analysis of busulfan (Bu) and deuterated internal standard in an LLOQ calibrator.

Interfering peaks were not detected under the described LC-MS/MS conditions. Representative chromatograms obtained are shown in Figure 1. 

#### 3.2.2. Matrix Effect and Extraction Recoveries

The matrix effect and IS normalized matrix effect were between 8 and 12%, and the extraction recovery was 90% (CV% < 15%).

#### 3.2.3. Calibration Curve and Range

**Figure 2:**
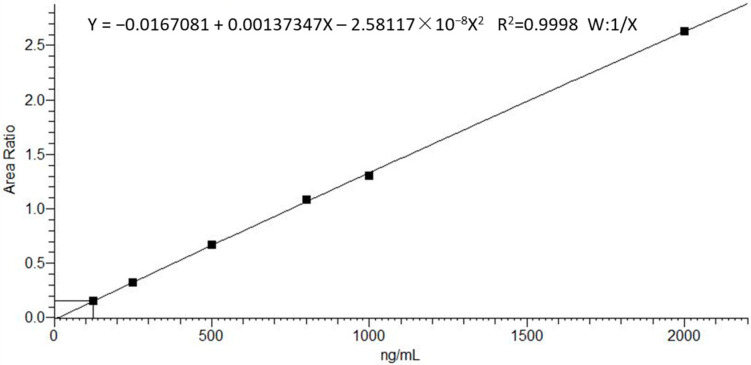
Six-point mean calibration curve of Bu in plasma (125–2000 ng/mL).

The LLOQ was 125 ng/mL for Bu. A weighted (1/x) linear regression model was used. The mean calibration curve statistics were Y = −0.0167081 + 0.00137347X − 2.58117 × 10^−8^X^2^ with R2 = 0.9998 for Bu in plasma (Figure 2). A linear relationship was obtained between the analyte peak area and the corresponding concentration for the entire concentration range (R2 = 0.99). The Bu concentration values obtained did not deviate significantly from the nominal values (±15%).

#### 3.2.4. Precision and Accuracy

Intra- and inter-assay precision (Table 3) and accuracy were within acceptable ranges (±15%).

#### 3.2.5. Carry Over

Carry-over was negligible. 

#### 3.2.6. Dilution Integrity

The dilution integrity results met the acceptance criteria for accuracy required by ICH guidelines M10.

#### 3.2.7. Stability

- *Freeze-thaw stability in matrix*

After the freeze/thaw and autosampler stability tests, no degradation was observed.

- *Stability in spiked sampled*

Long-term stability tests demonstrated that Bu was stable in spiked plasma stored at −20 °C and −80 °C after 4 weeks and at +4 ± 3 °C and at −20 °C for 24 h. Bu was found not to be stable in spiked samples stored at room temperature for 24 h, with percentage differences higher than 27%.

- *Autosampler stability*

Bu proved to be stable for 24 h after extraction if stored in the autosampler.

- *Stability of the analyte in whole blood*

Bu was proven to be stable in whole blood if stored at 4 ± 3 °C for 24 h, with percentage differences inside the acceptable ranges (13%). Conversely, Bu was not stable in whole blood when stored at room temperature for 24 h, with a percentage degradation higher than 38%.

- *Stability of the analyte in real samples*

Bu was not stable in patients’ plasma samples stored at room temperature for 24 h, with percentage differences higher than 33%. Bu was stable in patients’ plasma when stored at 4 ± 3 °C and at −20 °C, with percentage differences below 13%.

### 3.3. Analyses of Clinical Samples 

In line with ICH Guideline M10, to evaluate the incurred sample’s reanalysis precision, sixty plasma samples were tested in two different analytical runs. During the method development process, protein precipitation was initially performed by adding 150 µL acetonitrile to a 50 µL plasma sample (Protocol 2, paragraph 3.1). During the validation process, both QCs and real samples were analyzed in triplicate in 3 different analytical sessions. Although the results obtained for QCs were acceptable in terms of intra-day and inter-day reproducibilities (CV < 15%), those obtained on real samples ranged from 10% to 40% (mean = 17%). Two additional sample preparation protocols, including a higher volume of the organic phase, were then tested, obtaining an improvement of reproducibilities (10% and 4% for protocols 5 and 6, respectively), therefore meeting the criteria of ICH M10 guidelines. The main patients’ pharmacokinetic (PK) parameters obtained ranged within Bu’s narrow therapeutic window and are summarized in Table 4.

### 3.4. External Quality Assessment

Results were within the ranges of acceptance for all the samples tested.

## 4. Discussion

The availability of reliable and robust methods for Bu determination that are validated for clinical use is critical for improving therapeutic drug monitoring (TDM) practice. Despite the importance of this analysis, no certified method for in vitro diagnostics is available on the market; therefore, laboratories are forced to use their own laboratory-developed tests. Several analytical methods for the measurement of Bu in plasma have been previously published, including GC-MS and HPLC-UV [6,14,15,16,17], the latter being the most widely used since the 1980s. The bottleneck of HPLC methods is the lack of specificity in the detection method based on UV-VIS. Nowadays, LC-MS/MS is considered the gold standard for the quantitative measurement of small molecules. Protein precipitation is the most commonly used sample preparation protocol for therapeutic drug monitoring purposes. In fact, to quantitate a drug from a plasma sample, it is often necessary to break the bond between the drug and the plasma protein [22,23]. A number of LC-MS/MS methods for Bu quantification in human plasma, presenting heterogeneous sample preparation procedures, have been previously published [9,24,25,26,27,28,29]. Different precipitating agents, such as ACN/water [24], ACN [25,26,29], methanol [9,27], and ACN with 0.1% FA [28], with different plasma:organic phase ratios (from 1:2 to 1:6) were used, and different plasma volumes (from 50 µL to 200 µL) were adopted.

We initially tested a simple protein precipitation protocol using a plasma:organic phase ratio of 1:3 and obtained adequate precision and accuracy on QCs (±15%) but not on real samples. The use of ACN with 0.1% of formic acid in a plasma:organic phase ratio of 1:9 allowed us to also obtain acceptable reproducibility on real samples. This fact could be explained by enhanced protein precipitation, which could be obtained by the addition of a higher amount of organic phase, considering that the binding of Bu to plasma proteins ranges from 32 to 55 percent [30]. During the precipitation process, proteins are denatured, destroying their binding to the drug. Different protein precipitation techniques (e.g., organic solvents, acids, salts) are based on different protein precipitation patterns. Organic precipitants reduce the dielectric constant of plasma protein solution, facilitating electrostatic interactions between proteins [22]. The organic solvent surrounds proteins, minimizing hydrophobic interactions, making electrostatic interactions predominant and leading to protein aggregation. In contrast, acidic reagents lead to protein precipitation through the formation of insoluble salts [22]. For these reasons, we believe the adoption of protein precipitation protocols based on both organic solvent (ACN) and formic acid (AF) is most effective.

In this paper, we show the development of a robust and rapid LC-MS/MS method for Bu that was validated, for the first time, following the newest ICH guidelines M10 and tested on real samples derived from pediatric patients under treatment with Bu for a conditioning regimen before hematopoietic stem cell transplantation (HSCT). The analysis of stability on real samples allowed us to set up a robust protocol able to guarantee a reliable application to real-life conditions. Our results are in line with those obtained by Mürdter et al. [31], which tested the long-term stability of Bu in real samples stored at −20 °C for 3 months and short-term stability both at 4 °C and at room temperature for 6 days. Very importantly, stability test results revealed that samples for Bu measurement cannot be stored and transported at room temperature, and therefore, our protocol involves the immediate centrifugation and analysis of samples after collection; otherwise, refrigeration or freezing are highly recommended if samples are received from other centers. Another advantage of our method is the short chromatographic run (6.5 min), which allows for rapid turn-around times from the receipt of the last PK sample (6 h after Bu administration) to the reporting of results, allowing for a rapid answer to clinicians’ needs, in order to make next-dose recommendations in a timely fashion. Dose personalization is essential for high-dose Bu conditioning to reduce toxicity and improve clinical outcomes [8]. Our method is robust and fast to allow the delivery of the Bu concentration–time data result to accurately estimate patients’ PKs and make next-dose recommendations. Therefore, the method can be implemented by other laboratories to improve the current clinical practice of the related transplant centre.

## 5. Conclusions

In this paper, we report, for the first time, a LC-MS/MS method for Bu quantification in plasma validated according to ICH M10 (EMA) guidelines and suitable for the TDM-guided personalization of conditioning treatments with high-dose busulfan in pediatric patients undergoing HSCT.

## Figures and Tables

**Table 1:** Gradient phase concentrations (%*v*/*v*) during chromatographic run.

Time (min)	Phase A (%)	Phase B (%)
0.00	95%	5%
0.10	95%	5%
2.00	0%	100%
3.00	0%	100%
6.50	95%	5%

**Table 2:** Sample preparation protocols tested. ER% is extraction recovery %. CV% is the coefficient of variation percentage obtained on QCs low and high (*n*, replicates; (*n*) = 5).

Protocol	Sample Volume	IS Volume	Extraction Procedure	ER%	QC Low CV% (*n* = 5)	QC High CV% (*n* = 5)
1	50 µL	10 µL	150 µL MeOH	88%	2%	2%
2	50 µL	10 µL	150 µL ACN	90%	1%	1%
3	50 µL	10 µL	100 µL MeOH + 50 µL zinc sulphate 0.1 M	87%	4%	3%
4	50 µL	10 µL	100 µL ACN + 50 µL zinc sulphate 0.1 M	87%	5%	5%
5	50 µL	10 µL	440 µL ACN	89%	2%	2%
6	50 µL	10 µL	440 µL 0.1% *v*/*v* FA in ACN	90%	1%	1%

**Table 3:** Results of inter-day and intra-day accuracy and precision (expressed as CV%) assays (*n* = 5). SD is standard deviation.

Busulfan					
Inter-Day			Intra-Day		
	CV%	Accuracy		CV%	Accuracy
**LLOQ**	1%	12%	**LLOQ**	3%	11%
**QC low**	3%	−7%	**QC low**	5%	−4%
**QC medium**	2%	−4%	**QC medium**	4%	−2%
**QC high**	4%	−3%	**QC high**	5%	2%

**Table 4:** PK parameters (single I.V. dose) of Bu in 12 pediatric patients: constant elimination rate (K_el_); area under the plasma concentration–time curve from the time of dosing and extrapolated to infinity (AUC_inf_ expressed as mg/L × hour); terminal half-life (t_1/2_) in hours; average plasma drug concentration (ng/mL) at steady-state (C_ss_).

Patient	Kel	AUC_inf_ (mg/L × h)	T_1/2_ (h)	Css (ng/mL)
1	0.31	4.49	2.22	955.12
2	0.25	4.15	2.78	983.15
3	0.34	3.70	2.03	764.15
4	0.28	3.79	2.46	840.53
5	0.26	3.36	2.71	803.98
6	0.21	3.33	3.38	867.15
7	0.35	4.04	1.98	818.42
8	0.22	4.44	3.20	1130.46
9	0.33	2.90	2.07	606.61
10	0.40	3.60	1.74	696.00
11	0.23	2.9	3.01	696.95
12	0.33	2.79	2.11	578.63

## Data Availability

The data presented in this study are available from the corresponding author upon special request.

## Supplementary Materials

The following are available online at https://www.mdpi.com/article/10.3390/biomedicines11020530/s1, Figure S1: Chromatographic peaks obtained in spiked sample of Busulfan 2000 ng/mL with 3 different tested chromatographic columns: (1) Thermo Scientific™ Accucore™ Polar Premium column (50 mm × 2.1 mm, i.d. 2.6 µm, Thermo Fisher Scientific, Milan, Italy), (2) Hypersil GOLD aQ (50 × 2.1 mm, i.d. 1.9 µm, Thermo Fisher Scientific, Milan, Italy) and (3) Acquity UPLC BEH C18 (2.1 mm × 100 mm, i.d. 1.7 µm, Waters SpA, Milan, Italy). Table S1: Resolution, symmetry and peak classification parameters set in the peak suitability software evaluated for chromatographic column selection, Table S2: Results obtained from each cromatographic column tested during method development. Column 1: Thermo ScientificTM AccucoreTM Polar Premium column (50 mm × 2.1 mm, i.d. 2.6 µm, Thermo Fisher Scientific, Milan, Italy). Column 2: Hypersil GOLD aQ (50 × 2.1 mm, i.d. 1.9 µm, Thermo Fisher Scientific, Milan, Italy). Column 3: Acquity UPLC BEH C18 (2.1 mm × 100 mm, i.d. 1.7 µm, Waters SpA, Milan, Italy).
