# Supplementary material for: Development and Validation of a Novel LC-MS/MS Method for a TDM-Guided Personalization of HSCT Conditioning with High-Dose Busulfan in Children
Source: Biomedicines. 2023 Feb 11;11(2):530. doi: 10.3390/biomedicines11020530 (PMC9953620; doi:10.3390/biomedicines11020530)
Supplement: Supplementary file 1 [file biomedicines-11-00530-s001.zip › biomedicines-2127548-supplementary.pdf]

## SUPPLEMENTARY MATERIALS

**Supplementary Figure S1.** Chromatographic peaks obtained in spiked sample of Busulfan 2000 ng/mL with 3 different tested chromatographic columns: 1) Thermo Scientific™ Accucore™ Polar Premium column (50 mm × 2.1 mm, i.d. 2.6 µm, Thermo Fisher Scientific, Milan, Italy), 2) Hypersil GOLD aQ ( 50 x2.1 mm, i.d. 1.9 µm, Thermo Fisher Scientific, Milan, Italy) and 3) Acquity UPLC BEH C18 (2.1 mm × 100 mm, i.d. 1.7 µm, Waters SpA, Milan, Italy).

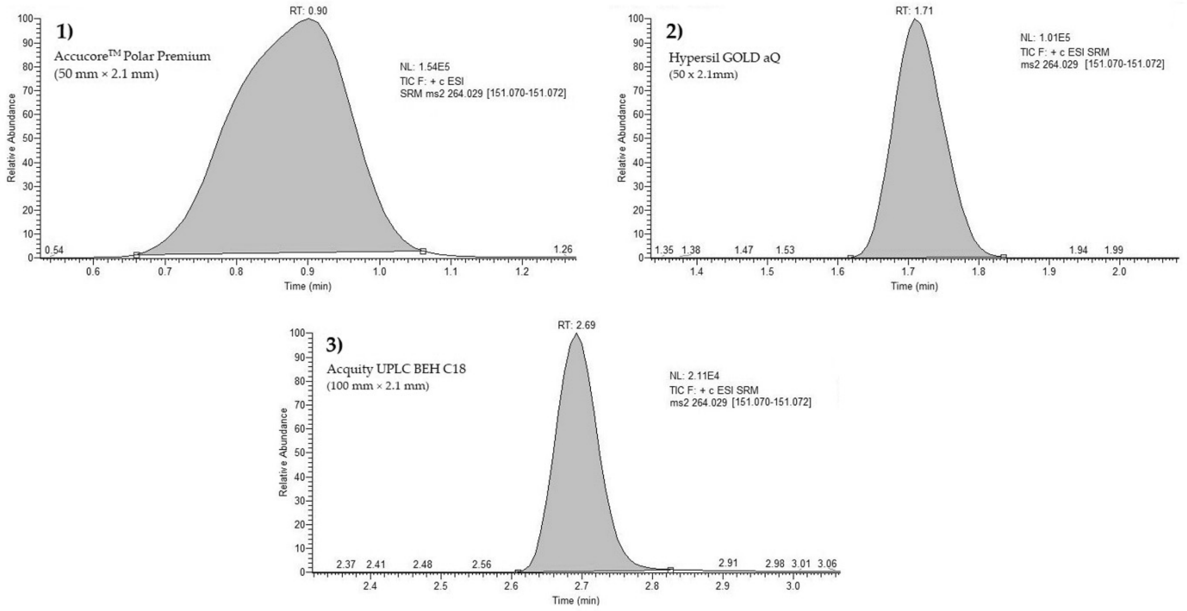


**Supplementary Table S1.** Resolution, symmetry and peak classification parameters set in the peak suitability software evaluated for chromatographic column selection.

| | | |
|---|---|---|
| **Resolution Parameters** | Resolution Threshold (%) | 90% |
| **Simmetry Parameters** | Peak height (%) | 50% |
| | Symmetry threshold (%) | 90% |
| **Peak classification Parameters** | Peak height (%) | 50% |
| | Min peak width (sec) | 1.80 sec |
| | Max peak width (sec) | 3.60 sec |
| **Detect tailing** | Peak height (%) | 10% |
| | Faileure threshold | 2.0 |
| **Detect column overload** | Peak height (%) | 50 % |
| | Faileure threshold | 1.5 |
| **Detect baseline clipping** | Number of peak for noise detection | 1.0 |
| **Detect minimum Signal-to-noise ratio** | Signal-to-noise ratio | 3 |

**Supplementary Table S2.** Results obtained from each cromatographic column tested during method development. Column 1: Thermo ScientificTM AccucoreTM Polar Premium column (50 mm × 2.1 mm, i.d. 2.6 μm, Thermo Fisher Scientific, Milan, Italy). Column 2: Hypersil GOLD aQ ( 50 x2.1 mm, i.d. 1.9 μm, Thermo Fisher Scientific, Milan, Italy). Column 3: Acquity UPLC BEH C18 (2.1 mm × 100 mm, i.d. 1.7 μm, Waters SpA, Milan, Italy).

| | **Column 1** | **Column 2** | **Column 3** |
|---|---|---|---|
| **Symmetrical** | Failed | Failed | Passed |
| **Resolution** | Failed | Passed | Passed |
| **Peak Width** | Failed | Failed | Failed |
| **Tailing** | Failed | Failed | Passed |
| **Column Overload** | Failed | Passed | Passed |
| **Baseline Clipping** | Failed | Passed | Passed |
| **Signal-to-noise ratio** | Failed | Passed | Passed |
| **Concave** | Failed | Passed | Passed |
| **Saturation** | Failed | Passed | Passed |
